# Genomic and metabolic profiling of two tomato contrasting cultivars for tolerance to *Tuta absoluta*

**DOI:** 10.1007/s00425-023-04073-8

**Published:** 2023-01-28

**Authors:** Daniela D’Esposito, Anna Guadagno, Ciro Gianmaria Amoroso, Pasquale Cascone, Gabriele Cencetti, Marco Michelozzi, Emilio Guerrieri, Maria Raffaella Ercolano

**Affiliations:** 1 Department of Agricultural Sciences, University of Naples Federico II, 80055 Portici, NA Italy; 2 Institute for Sustainable Plant Protection, National Research Council of Italy, 80055 Portici, NA Italy; 3 Institute of Biosciences and Bioresources, National Research Council of Italy, 50019 Sesto Fiorentino, FI Italy

**Keywords:** Genetic variants, Leaf miner, Plant defense, Solanum lycopersicum, Volatile organic compounds

## Abstract

**Main conclusion:**

Dissimilar patterns of variants affecting genes involved in response to herbivory, including those leading to difference in VOC production, were identified in tomato lines with contrasting response to *Tuta absoluta*.

**Abstract:**

*Tuta absoluta* is one of the most destructive insect pest affecting tomato production, causing important yield losses both in open field and greenhouse. The selection of tolerant varieties to *T. absoluta* is one of the sustainable approaches to control this invasive leafminer. In this study, the genomic diversity of two tomato varieties, one tolerant and the other susceptible to *T. absoluta* infestation was explored, allowing us to identify chromosome regions with highly dissimilar pattern. Genes affected by potential functional variants were involved in several processes, including response to herbivory and secondary metabolism. A metabolic analysis for volatile organic compounds (VOCs) was also performed, highlighting a difference in several classes of chemicals in the two genotypes. Taken together, these findings can aid tomato breeding programs aiming to develop tolerant plants to *T. absoluta*.

**Supplementary Information:**

The online version contains supplementary material available at 10.1007/s00425-023-04073-8.

## Introduction

Tomato (*Solanum lycopersicum* L.) is the most valuable vegetable crop widely grown around the world and consumed in both fresh and processed forms. Biotic factors, e.g. insects and pathogens, are major threats for the tomato production. In recent years, the South American tomato pinworm *Tuta absoluta* (Meyrick) (Lepidoptera: Gelechiidae) emerged as one of the most devastating insect pests for tomato crops all over the world regardless the type (greenhouse, open field) or the cycle of cultivation (spring–summer, autumn–winter) (Desneux et al. 2010, 2011; Cocco et al. 2013; Campos et al. 2017). Larvae of *T. absoluta* can destroy the tomato canopy by producing mines in the leaves, stems and buds, and burrows into fruits that significantly affect yield and tomato fruit quality (Cocco et al. 2013). Chemical control methods against *T. absoluta* are becoming less effective and sustainable due to the feeding habits of the larvae, the number of generations completed during the cultivation and the increase of resistance of this pest to the most used chemical active substances (Moreno et al. 2012; Cocco et al. 2013; Biondi et al. 2018). Therefore, there is a growing demand for sustainable approaches to control this invasive pest that includes the biological control (performed by predators and parasitoids) and the selection of tolerant varieties (Pérez-Hedo and Urbaneja 2016; Giorgini et al. 2018; Kayahan et al. 2018).

In a previous study, we showed a global view of transcriptome changes of two tomato cultivars, one tolerant and one susceptible to *T. absoluta* (D’Esposito et al. 2021). The tolerant line showed a four-time lower number of eggs and mines per plant compared to the susceptible genotype. Even the number of damaged fruits was much higher in the susceptible genotype compared to the tolerant genotype (33% and 6.5%, respectively). We attributed these differences to both, the glandular and non-glandular trichomes, leaf density and to the re-modulation of the transcriptional response. Differentially expression of genes involved in leaf development, structural meristem formation and photosynthesis, as well as genes involved in defense response such as receptor-like kinases (RLKs), MAPKs, calcium‐dependent protein kinases (CDPKs) and transcription factors (TFs) (D’Esposito et al. 2021). In addition, transcript alteration of key players related to the recognition of the insect, the signaling mediated by jasmonic acid (JA), the trichomes development and the terpenes synthesis in both the tolerant and susceptible interactions have been highlighted. The phenotypic diversity that emerged between the two cultivars is coupled to genetic loci that need to be explored at genomic level.

Single nucleotide polymorphisms (SNPs) and insertions/deletions (InDels) are responsible for the genetic diversity existing among individuals and can cause a phenotypic diversity for many traits, including the tolerance to various biotic factors (Morgil et al. 2020). For example, non synonymous SNPs in coding regions can alter the amino acid sequence of a protein determining a biological change (Yates and Sternberg 2013). In addition, SNPs falling in regulatory sequences can affect plant gene expression (Morgil et al. 2020). Therefore, the detection of SNPs/InDels in gene transcripts can be relevant for functional analysis because they can be used to characterize variants affecting protein role.

The aim of this work was to investigate the genomic and metabolic variation that contributes to the phenotypic diversity observed in tomato tolerant and susceptible genotypes (BR221 and PS650) to *T. absoluta*, characterized at trascriptomic level in our previous work (D’Esposito et al. 2021). This study revealed also clear differences in the degree of infestation and trichomes density between the two genotypes. The transcriptomic sequencing data obtained in the former work represent a valuable resource for the exploitation and the characterization of SNP and InDel polymorphisms in tomato tolerant and susceptible genotypes to *T. absoluta*. Genes carrying small variants were investigated for their potential effect within the pathway of response to biotic stress. In addition, a metabolic profile was performed on the tolerant and the susceptible genotypes to analyze VOCs released constitutively or upon attack by *T. absoluta.* The discovery of genomic and metabolic variation can increase our knowledge about tomato genetic diversity in response to pest attack and can provide a better understanding of tolerance process useful to support crop breeding programs.

## Materials and methods

### Variant identification and annotation

The raw reads from Illumina RNA-Seq produced in our previous work (D’Esposito et al. 2021) were cleaned and filtered for quality. The high-quality reads were aligned to tomato genome reference SL3.0 by A.I.R platform (https://transcriptomics.sequentiabiotech.com). The BAM files generated for tolerant and susceptible genotypes were used for SNP calling using BCFtools (Li 2011; https://samtools.github.io/bcftools/bcftools.html). SNPs were filtered for quality using the following parameters: allele frequency (AF) higher than 0.75, minimum quality (QUAL) and minimum genotype quality (GQ) equal to 30, depth between 5 and 100 and minimum mapping quality (MQ) equal to 20. The program SnpEff (Cingolani et al. 2012; http://SnpEff.sourceforge.net/) was used to annotate private variants for each genotype based on their genomic locations and to predict variant effects.

### Gene functional annotation

The functional annotation of genes affected by variants with high (modification of gene products), moderate (missense variations due to changes amino acid codons) and modifier (non-coding variants or variants affecting non-coding genes) effect was performed using MapMan (Thimm et al. 2004). Assignment to MapMan classes was performed using the Mercator pipeline. Tomato pathway annotations for genes with variants were retrieved by the Plant Metabolic Network Database (Hawkins et al. 2021), TomatoCyc version 5.0.1 based on ITAG3.2 annotation.

### Infestation experiment

The initial strain of *T. absoluta* was collected in 2017 in tomato greenhouses located in Battipaglia (Salerno, Italy). It was continuously reared at the Istituto per la Protezione Sostenibile delle Piante (IPSP) inside bug dorms^®^ isolators on tomato plants (cultivar ‘San Marzano nano’) at the following conditions: temperature of 24 ± 2 °C, relative humidity (RH) of 65 ± 5%, and photoperiod of 16L:8D.

The tomato genotypes BR221 (T = tolerant) and PS650 (S = susceptible) were grown in a glasshouse under the following conditions: temperature of 24 ± 2 °C, relative humidity (RH) of 65 ± 5%, and photoperiod of 16L:8D. Five-week-old plants, with 4–6 completely expanded leaves and a height of 18 cm, were placed into a single mesh cage (60 × 60x180 cm; Vermandel, Hulst, The Netherlands). Three to five days old mated females of *T. absoluta* were released into the cage keeping a 1:1 ratio between them and the plants. After two days (oviposition period), the females were removed from the cage by an insect aspirator. Plants were kept for 12–14 days in the same conditions reported above until the larvae hatched. Eleven replicates for each uninfested plants and plants infested by larvae were individually placed inside a 20-L glass jar for a 3-h volatile extraction from the headspace of those plants.

### Volatile analysis

The volatile compounds were collected in Tenax traps and stored at − 20 °C for later volatile profiling by means of gas chromatography. After trapping on Tenax (30 mg) and carboxen (30 mg) packed tubes, the samples were analyzed by CIS4–TDU–GC/MS. Gerstel TDU (Gerstel, Mülheim, Germany) was heated at 300 °C for 7 min under a helium stripping flow of 30 mL min^−1^. The TDU unit was directly assembled over the PTV injector (CIS4, Gerstel) with a liner-in-liner coupling, which eliminates the carryover effect and analyte loss. During this stage, the CIS4 was cooled to − 20 °C by computer-controlled liquid CO_2_ pulsed flow. After cryo-trapping on a Tenax packing liner, the PTV was quickly ramped to 260 °C for desorption and the analyte was transferred to CIS4. An Agilent 7890 GC equipped with a 5975 MSD was used for the analysis, all from Agilent Tech (Palo Alto, CA, USA). Helium was used as the carrier gas, and the flow was kept constant at 1.2 mL/min. The chromatographic settings were as follows: injector in splitless mode set at 260 °C, J&W Innowax column (50 m, 0.20 mm i.d., 0.4 µm df); oven temperature program: initial temperature 40 °C for 1 min, then 10 °C min^−1^ increase until 130 °C, then 5 °C min^−1^ increase until 210 °C, then 20 °C min^−1^ increase until 260 °C, hold time 3 min. The mass spectrometer was operating with an electron ionization of 70 eV, in scan mode in the m/z range 29–330, at three scans s^−1^. The deconvoluted peak spectra obtained by Agilent Mass-Hunter software were matched against the NIST 11 spectral library for tentative identification. Kovats’ retention indices were calculated for further compound confirmation and compared with those reported in the literature for the chromatographic column used. Authentic standards were also injected to confirm compound identity.

## Results

### Gene variant discovery and chromosomal distribution

**Fig. 1:**
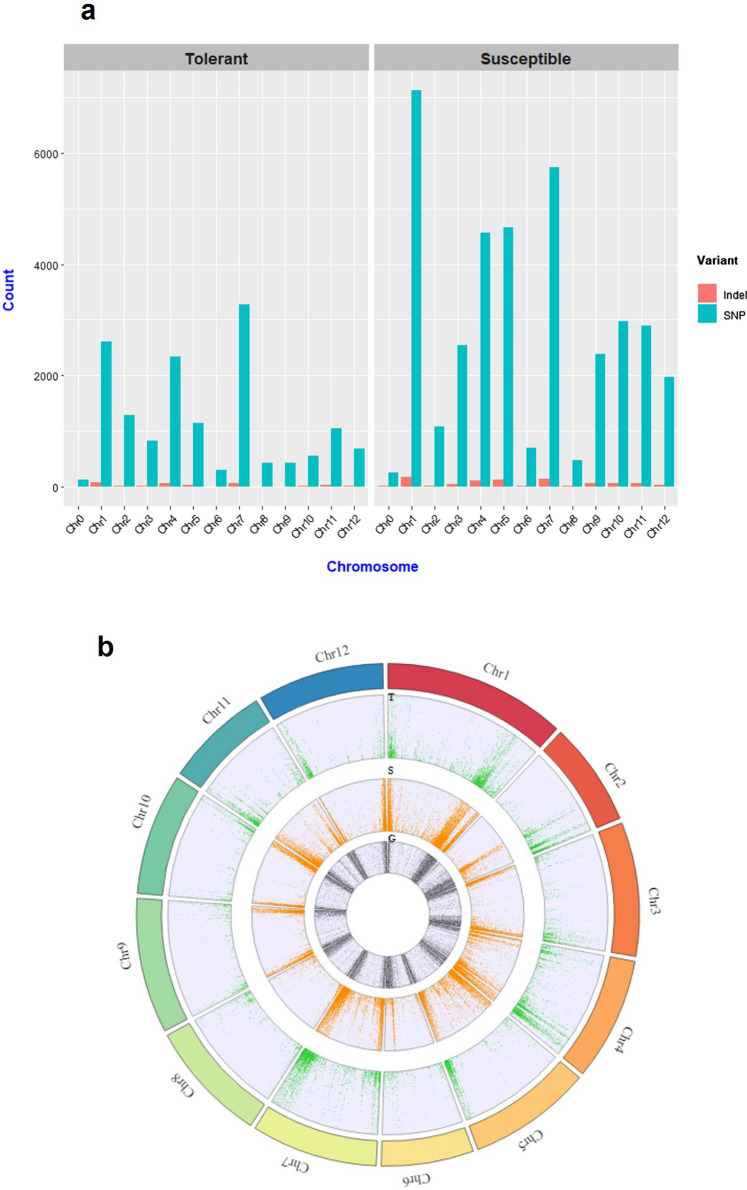
Variants distribution across the tomato genome. **a** SNPs and InDels count along the tomato chromosomes in tolerant and susceptible genotypes. **b** Circos plot of SNPs density along tomato chromosomes. From the outer ring to the inner ring: tomato chromosomes, SNP density distribution for tolerant genotype (T, in green); SNP density distribution for susceptible genotype (S, in orange) and the gene distribution (G, in gray)

A total of 15,031 and 37,385 high-quality homozygous SNPs and 302 and 845 homozygous InDels were detected in T and S, respectively (Supplementary Table S1, Table S2, Table S3, Table S4). For each chromosome the SNPs and InDels count (Fig. 1a) showed a higher number of SNPs in S, except for the chromosome 2, where T showed a slightly greater number (1285 SNPs in T and 1086 in S), and the chromosome 8 where the SNPs number was roughly the same in both genotypes (432 SNPs in T and 469 in S). For both genotypes the number of InDels detected was much lower with respect to SNPs and also in this case it was higher in S for all the chromosomes. The chromosome with the largest number of variants was the chromosome 1 (the longest chromosome) in S and the chromosome 7 in T, respectively.

**Fig. 2:**
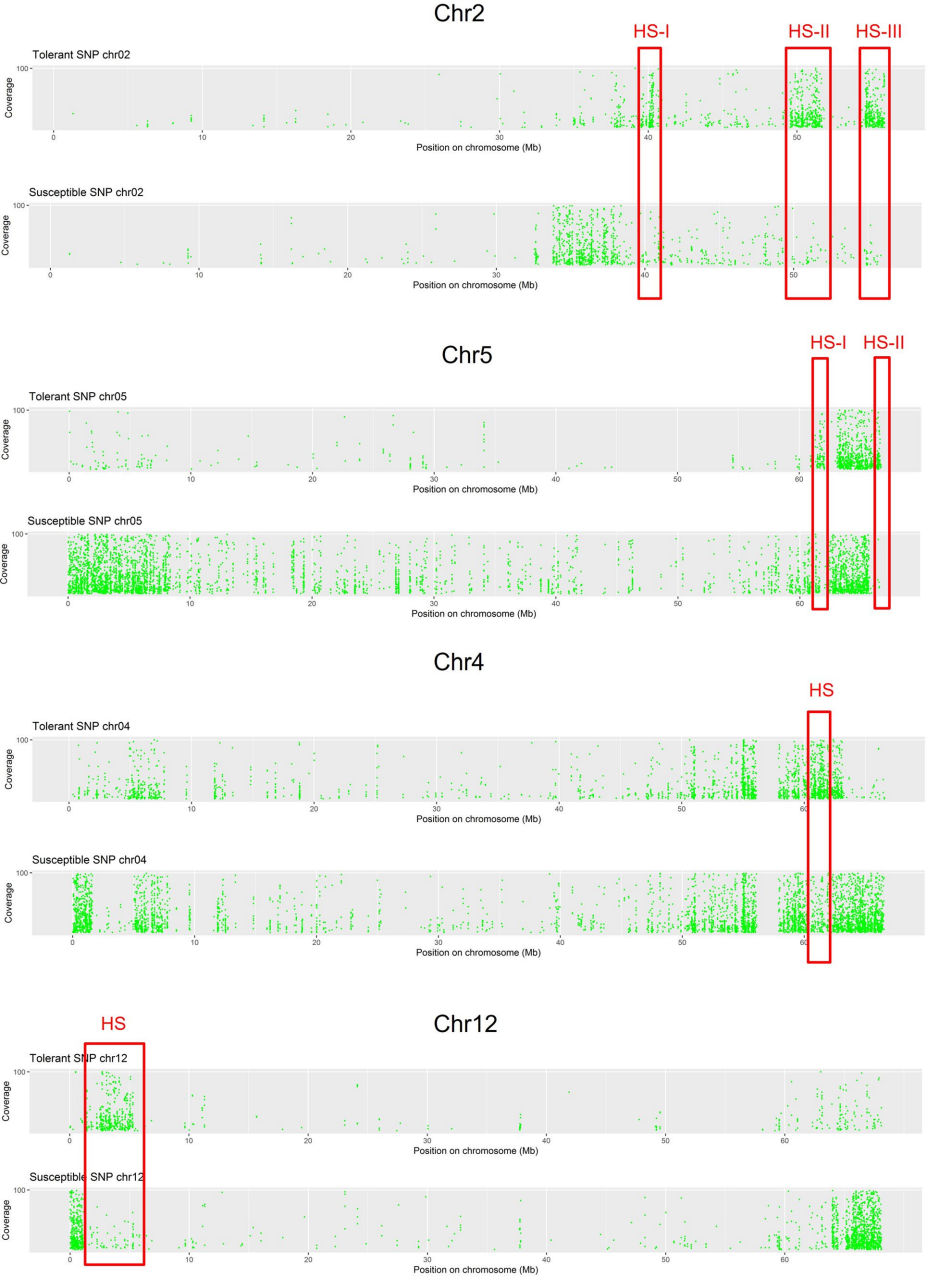
SNPs hotspots. SNPs density in chromosomes with evident dissimilar patterns of variants between tolerant and susceptible genotypes, in comparison to the reference genome. Red boxes indicate dissimilar pattern of variants. *HS* = variant hotspot

In addition, we noticed that T displayed regions with SNPs hotspots (HS) on some chromosomes, in which, on the contrary S showed a low number of variants (Fig. 1b, Fig. 2). In detail, in T genotype the chromosome 2 had a higher SNPs density in three regions ranging from 39–41 Mb, 49–52 Mb and 54–56 Mb, respectively. On the chromosome 4 a HS was identified between 60–62 Mb, on the chromosome 5 two HS were located in the distal region and finally, on chromosome 12 a HS was identified in the region ranging from 2 to 6 Mb (Fig. 2, Supplementary Table S5). Other divergent chromosome regions, although to less extent, were observed on chromosome 3, 7, 8, 9, 11 (Supplementary Table S5).

### Genotype-specific SNPs and InDels identification and annotation

To better identify causal variants that underlined tomato genotype-specific *T.absoluta* response, we focused on genotype-private variants, observed in a specific genotype (Table 1). In particular, private T variants included 8761 SNPs and 250 InDels (148 insertions and 102 deletions), while S displayed 31,116 SNPs and 793 private InDels (466 insertions and 327 deletions). Looking at the distribution of private variants along the chromosomes the T genotype showed a slightly higher number of private SNPs on the chromosome 2 (1146) than S (947) as well as a similar number of InDels (16 InDels in T and 19 in S).

**Table 1:** Summary of genotype-private variants (SNPs and InDels) identified along the tomato chromosomes

Chromosome	Tolerant (T)		Susceptible (S)	
	SNP	InDel	SNP	InDel
Chr0	74	2	196	5
Chr1	1299	60	5816	160
Chr2	1146	16	947	19
Chr3	533	13	2261	42
Chr4	1322	45	3556	97
Chr5	730	21	4256	116
Chr6	173	4	568	13
Chr7	1464	53	3943	123
Chr8	271	3	309	6
Chr9	290	1	2254	58
Chr10	345	5	2766	57
Chr11	544	19	2387	63
Chr12	570	8	1857	34
Total	8761	250	31,116	793

**Fig. 3:**
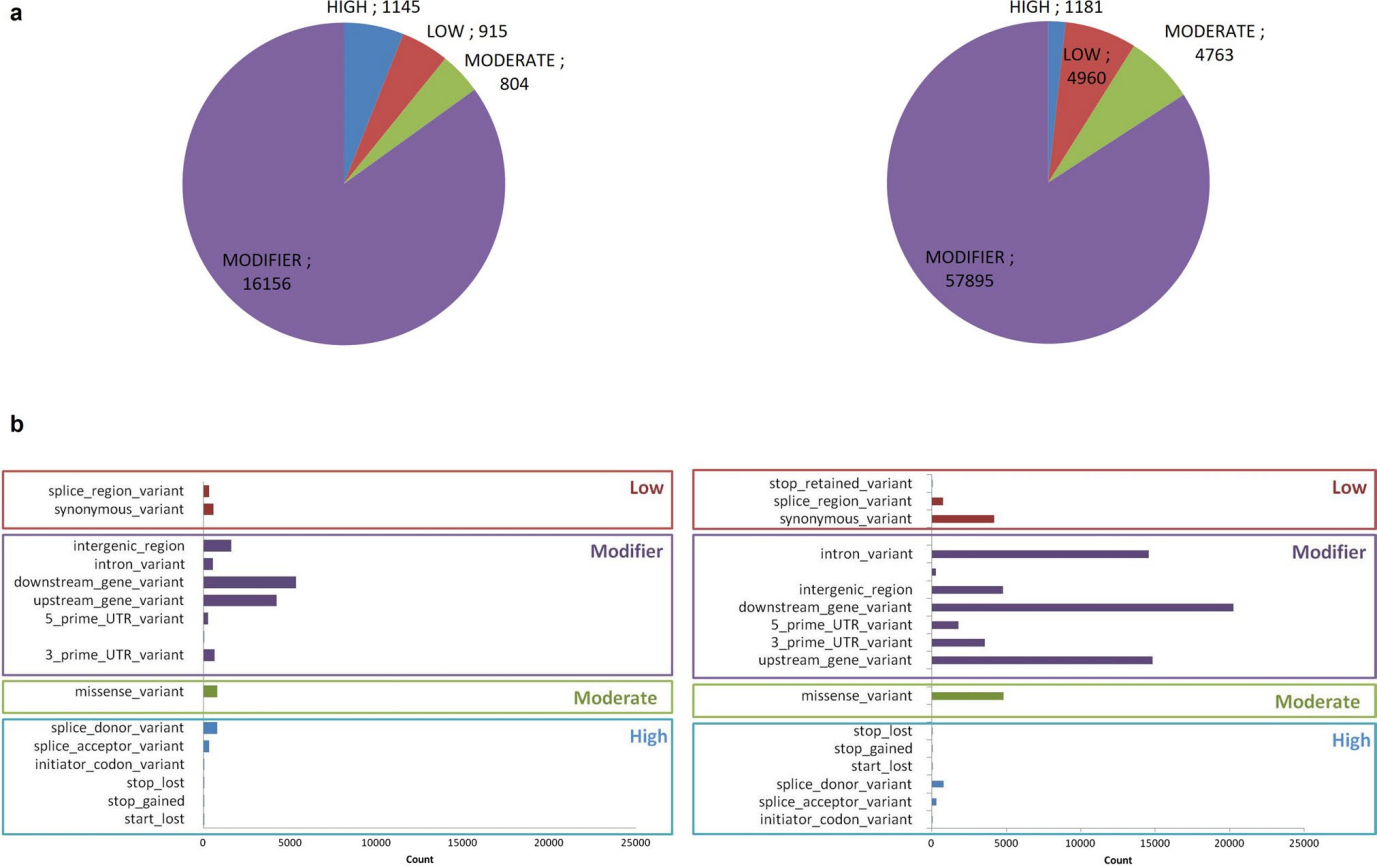
SNP annotation and prediction of variant effects. **a** Number of SNP classified as high, moderate, modifier and low effect in tolerant (on the left) and susceptible (on the right). **b** Sub-classification of variants based on effects in tolerant (on the left) and susceptible (on the right)

Private variants for both genotypes were annotated for their impact on the functionality of the genes and encoded proteins by classification in four categories (high, moderate, modifier, low). Figure 3a shows the distribution of SNP variants in the four categories, whilst InDels effect annotation is reported in Supplementary Fig. S1.

The vast majority of variants was classified in the “modifier” category, including downstream gene variant, intron variant and upstream gene variant, whilst the less abundant corresponded to the “high” variation effect. A good number of missense variations showed a moderate effect (Fig. 3a, b). The most abundant variants with a high impact were the “splice donor,” (796 and 780, in T and S genotypes, respectively) followed by “splice acceptor variants” (320, T and 329, S) (Fig. 3b).

### Genes involved in biotic response affected by potential functional variants

To explore the potential variations with effect at protein and transcript level, we focused on SNPs and InDels, with high, moderate and modifier effect in each tomato genotype. In T genotype 6294 genes were affected by SNPs and or InDels classified in one, two or all the three above mentioned classes, while 12,668 were found in the S. It is worth noting that, although the variants are different between the two tomato genotypes, they can affect common genes.

**Fig. 4:**
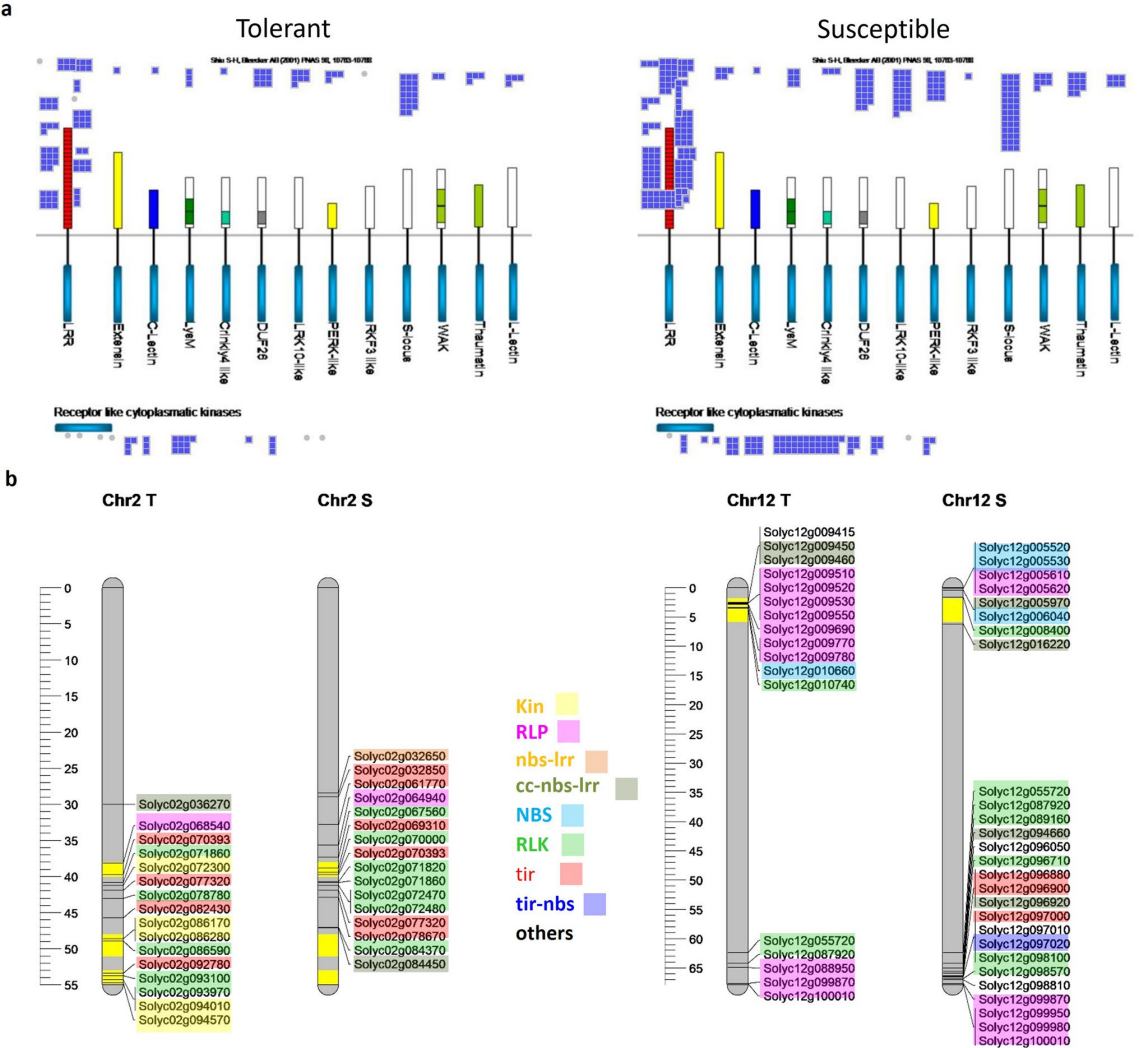
Mapping and distribution of receptor kinases. **a** Mapman classification of receptor kinases in tolerant (on the left) and susceptible (on the right) genotypes. **b** Localization of receptors with variants on the chromosome 2 and 12. *T* tolerant, *S* susceptible. The scale on the left of the chromosomes is expressed in megabases (Mb). Genes belonging to different receptor classes are marked with rectangles of different colors. The variants hotspot (HSs) regions identified in T are highlighted in yellow. For better comparison HSs are reported for S too. *Kin* kinase, *RLP* receptor-like proteins, *nbs* nucleotide-binding site, *lrr* leucine-rich repeat, *cc* coiled-coil, *RLK* receptor-like kinases, *tir* toll interleukin 1 receptor

Both genotypes showed variants in genes related to stimulus perception, hormone metabolism, cell wall, signaling and secondary metabolism. Genes coding for receptor-like kinases (RLKs) and receptor-like proteins (RLPs) and harboring variants were identified in both genotypes (Fig. 4a). In T genotype a higher number of RLP with variants was located within the HS region of the chromosome 12 (Fig. 4b). RLKs were also present in the HSIII on the chromosome 2. On the same chromosome, variants in genes belonging to kinase/phosphatase signaling were also found in T genotype, including the phosphatase Solyc02g070260. Other genes affected by variants in T genotype were the leucine-rich repeat receptor-like (Solyc04g076460) as well as the kinase SlSERK3B (Solyc01g104970) and two genes coding for cyclic nucleotide-gated channels (Solyc02g086990, Solyc02g088560).

The prosystemin (Solyc05g051750) had variants with high effect in T genotype while S showed variants with modifier effect. Within JA biosynthesis, common and genotype-specific variants were identified (Supplementary Fig. S2). T-specific genes included two genes located in the HS of the chromosome 12 and HS-III on chromosome 2 (i.e. Solyc12g011040 and Solyc02g093140). In addition, T-specific variants also affected the lipoxygenase D on chromosome 3 (Solyc03g122340, TomLoxD). On the other hand, S genotype showed variants in LOXC.

T genotype also showed a higher number of fatty acid beta-oxidation multifunctional proteins with variants, including Solyc12g007170 in HS at the beginning of the chromosome 12. In this region, T-specific variants were also localized in subtilisin-like proteases (Solyc12g011140, Solyc12g011213). On the chromosome 2, T showed specific variants in Solyc02g094040, ortholog to Arabidopsis *MPL1*, *MYZUS PERSICAE-INDUCED LIPASE 1* (At5g14180) and in genes involved in cell wall metabolism, including a glucan endo-1,3-beta-glucosidase (Solyc02g069700), four CASP-like proteins, three cobra proteins (Solyc02g089115, Solyc02g089120, Solyc02g089130). Hydroxyproline-rich glycoproteins (Solyc04g076410, Solyc04g074165) and rhamnogalacturonate lyases (Solyc04g076630, Solyc04g076640, Solyc04g076650, Solyc04g076660) (Deepak et al. 2007). Among genes with S-specific variants there was the histone-lysine N-methyltransferase, H3 lysine-9 specific *SUVH1*, Solyc10g077070. Common genes affected by variants in both genotypes include an another subtilisin-like protease (Solyc01g087850) affected by missense variation.

Eight transcription factors (TF) with a role in biotic stress response (Table 2), showing variants in T genotype, were located on chromosome 2: Solyc02g037530, an auxin response factor 8B; two zinc finger proteins LSD1 (Solyc02g078270 and Solyc02g069720); three WRKY including Solyc02g094270, Solyc02g088340, Solyc02g093050; *MYB-SlMIXTA*-like (Solyc02g088190) and an AP2/EREBP TF (Solyc02g093130). An HD-Zip TF (Solyc08g066500 and *ULTRAPETALA* (Solyc12g010755), also showed variants in T genotype. Conversely, S-specific variants affected a *Woolly* gene (Wo), a *HOMEODOMAIN GLABROUS2* gene (Solyc12g005830), a basic helix-loop-helix (BHLH) transcription factor (Solyc09g083360), the *SCL3* (scarecrow-like 3) Solyc12g099900 and the *CUTIN DEFICIENT2* (Solyc01g091630).

**Table 2:** Variants affecting transcription factors (TFs) in Tolerant (T) and Susceptible (S) genotypes

Genotype	Gene ID	Functional description	Effect
Tolerant	Solyc02g088190	MYB-related transcription factor	Moderate (1) Modifier (1)
	Solyc02g037530	Auxin response factor 8B	High (1)
	Solyc02g078270	LSD1 zinc finger family protein	Modifier (1)
	Solyc02g069720	Zinc finger protein LSD1	Modifier (12) High (1)
	Solyc02g093130	AP2/EREBP transcription factor_1	Modifier (12)
	Solyc02g094270	WRKY transcription factor	Modifier (1)
	Solyc02g088340	WRKY transcription factor 3	Modifier (1)
	Solyc02g093050	WRKY transcription factor 8	Modifier (5)
	Solyc08g066500	Homeobox-leucine-zipper protein	High (1)
	Solyc12g014210	RNA-binding (RRM/RBD/RNP motifs) family protein-	Modifier (13)
Susceptible	Solyc02g080260	Woolly	Moderate (1)
	Solyc09g083360	Basic helix-loop-helix (BHLH) family transcription factor	Moderate (2) Modifier (2)
	Solyc12g005830	Homeobox-leucine-zipper family protein	Modifier (2)
	Solyc12g099900	SCL3 (scarecrow-like 3)	Modifier (3)
	Solyc01g091630	cutin deficient 2	Modifier (2)

The table shows information about the genotype, the gene ID, the gene functional description and the variant effect. In parentheses is showed the number of variants annotated with a specific effect

### Identification of genes with variants involved in volatile organic compounds production

**Fig. 5:**
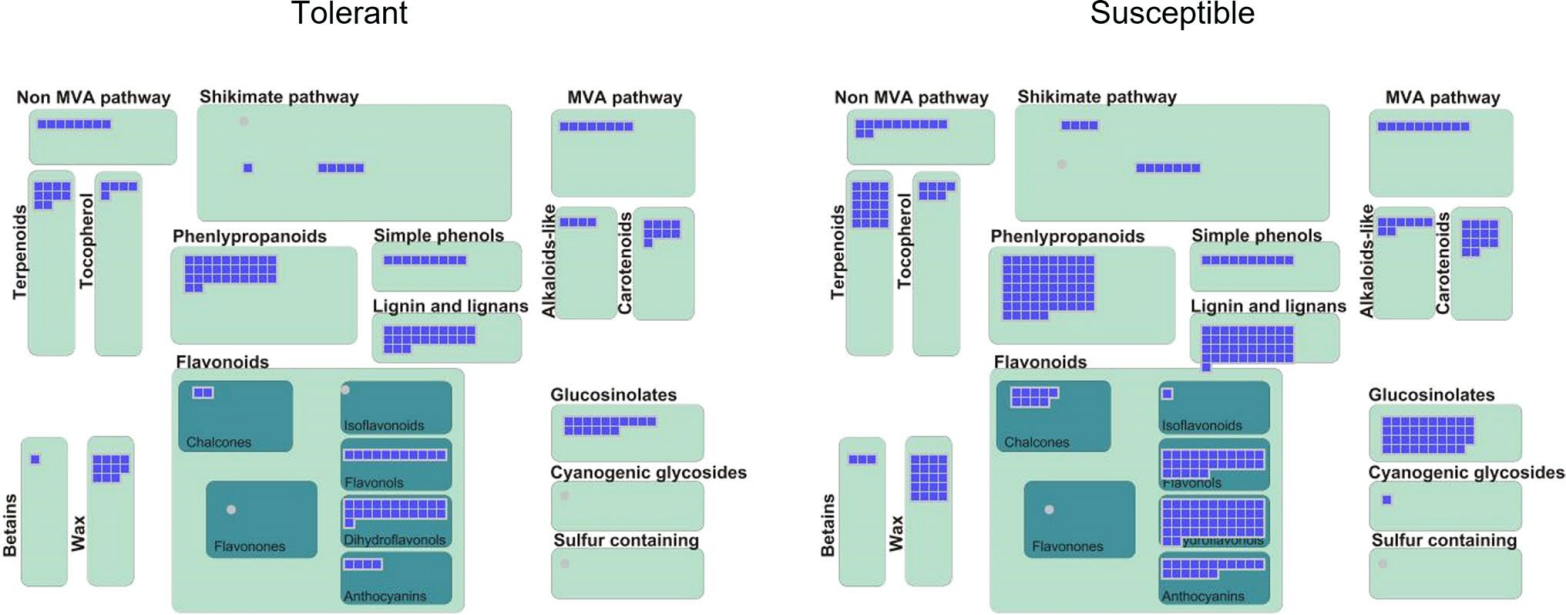
Overview of genes with variants mapping to secondary metabolism in Tolerant and Susceptible tomato genotypes. Each blue square corresponds to a gene

In both genotypes many genes with variants were involved in the production of secondary metabolites (Fig. 5). In the phenylpropanoid metabolism, T genotype displayed variants affecting four genes coding for 4-coumarate—CoA-ligases, while only one was affected in S (Supplementary Fig. S3). One of two genes coding for caffeoyl-CoA *O*-methyltransferase (CCoAMOT) was affected by variants in both genotypes (Solyc04g063210), while the second gene (Solyc02g093230) had variants only in T.

Genes involved in acylsugars biosynthetic pathway and acylsugars transport, were also affected by variants. Solyc03g121540, encoding for ACYLSUCROSE FRUCTOFURANOSIDASE 1 (ASFF1) and Solyc02g093180, encoding for an acyltransferase showed variants in T genotype (Supplementary Fig. S4). In addition, two genes with specific variants in T genotype (Solyc02g061740 and Solyc02g061750) and one with variants in both genotypes (Solyc04g025450), involved in the biosynthesis of acylsugar isoC5-CoA acyl chain precursor were found. In S genotype variants with impact on the ABC transporter Solyc03g005860 were identified.

**Fig. 6:**
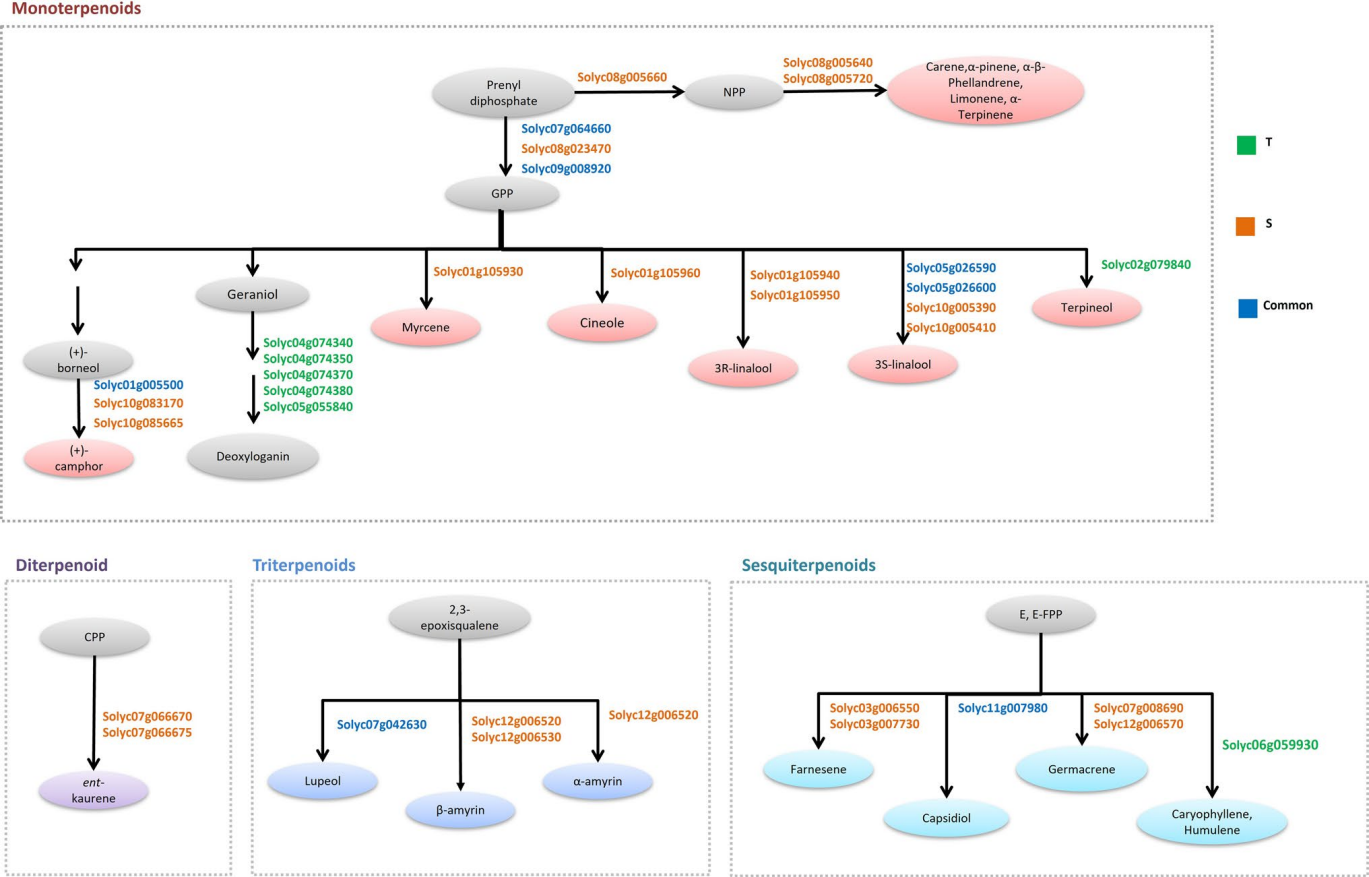
Genes affected by variants in the synthesis of terpenoids. Tolerant-specific genes, susceptible-specific genes and common genes are marked in green, orange and blue, respectively. *T* tolerant; *S* susceptible

Exploring the nucleotide variation in terpene synthase genes (TPS) along all the genome, several variants were identified in both genotypes of which four were commons, three T-specific and 11 S-specific (Fig. 6). Monoterpenes, diterpenes, triterpenes and sesquiterpenes pathways were analyzed.

Both genotypes showed genes with variants in camphor and linalool biosynthesis (Fig. 6). On the chromosome 2 of the T genotype, unique variants were identified in the terpene synthase Solyc02g079840 involved in the (−)-α-terpineol biosynthesis. Variants affecting five 7-deoxyloganetin glucosyltransferases (Solyc04g074340, Solyc04g074350, Solyc04g074370, Solyc04g074380, Solyc05g055840), located in HSs on chromosome 4 and 5, were also identified in T genotype. Differences were observed in the pentacyclic terpene biosynthesis leading to the production of lupeol and amyrin, due to two S-specific genes with variants (Solyc12g006520, Solyc12g006530) and one common to both genotypes (Solyc07g042630). A sesquiterpene synthase with T-specific variants, involved in the production of caryophyllene, was identified (Fig. 6).

**Fig. 7:**
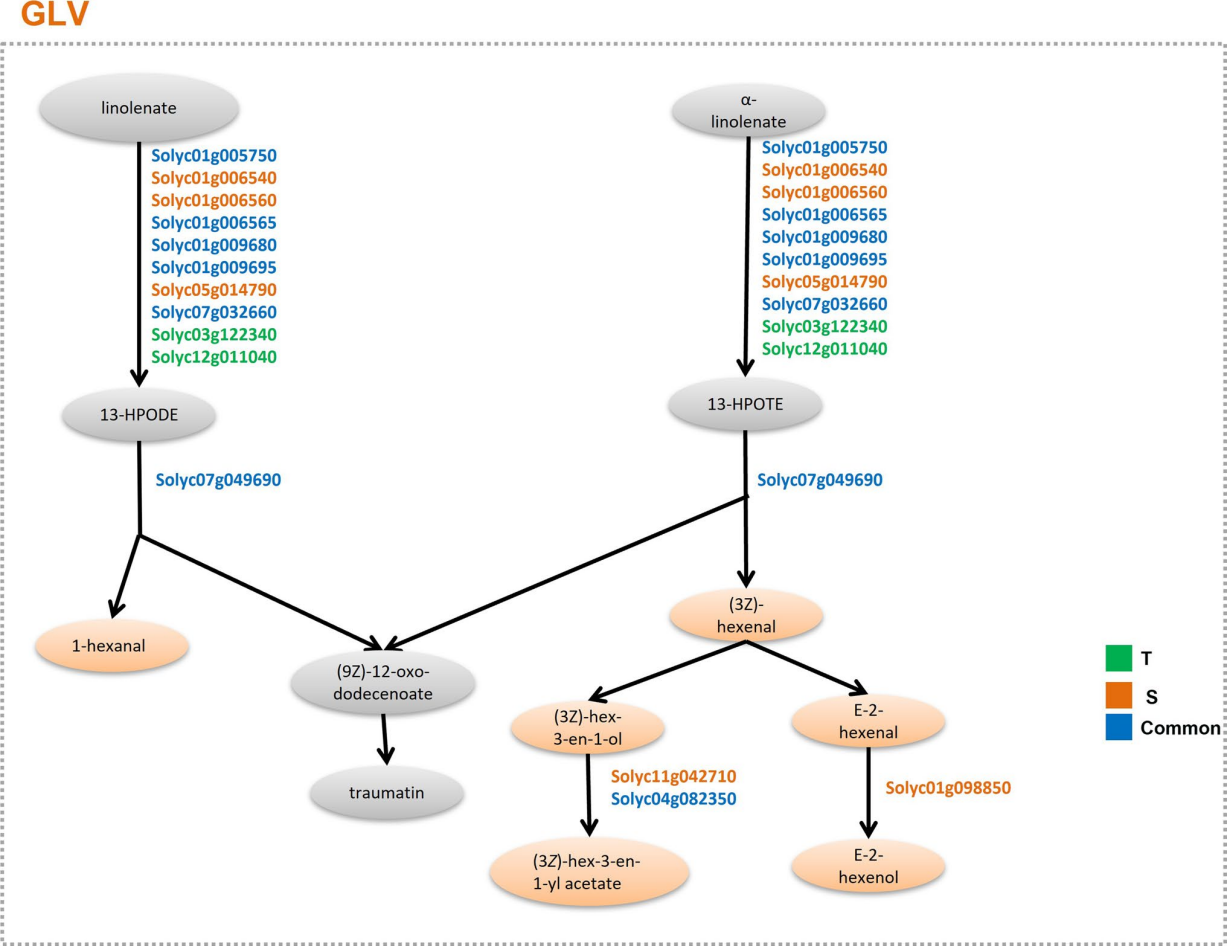
Genes affected by variants in the synthesis of green leaf volatiles (GLV). *T* tolerant; *S* susceptible

Regarding the synthesis of fatty acid derivatives green leaf volatiles (GLV), 9 and 12 genes with variants were identified, respectively in T and S genotypes (Fig. 7). In addition, two genes involved in the detoxification of reactive carbonyls in chloroplasts showed variants in S genotype.

Finally, two genes (Solyc02g069920, Solyc02g069925) involved in the production of 1,4-dihydroxy-2-naphthoate I, were polymorphic in T genotype. By contrast, the S genotype showed variants in a gene (Solyc01g108720) involved in methylsalycylate degradation.

### Volatile phenotyping

A total of 18 VOCs were identified through GC-MS analyses in the headspace collection from uninfested plants (T and S genotypes) and plants infested by *T. absoluta* larvae (T and S genotypes) belonging to different chemical classes and to different biosynthetic pathways (Table 3). In detail, we recovered: eleven monoterpenoid from the mevalonic acid (MVA) or the methylerythritol phosphate (MEP) pathways; two aldehydes and one alcohol as fatty acid derivatives; four benzenoid compounds from shikimate/phenylalanine pathway (Table3). Overall, all plants released blends composed by similar compounds, but in different proportions. In particular, the composition of the blend varied according to the genotype and in response to larvae infestation. For example, when uninfested, the T genotype showed a higher level of γ-terpinene and δ3-carene while the S a higher level of camphene, α-phellandrene, eucalyptol, (E)-2-Hexenal and cis-3-hexenol. Conversely, when infested with *T. absoluta* larvae, the T genotype showed a significant increase in virtually all identified volatile compounds.

**Table 3:** The mean amounts (ng g^−1^ fresh weight 3 h^−1^ ± SE, *n* = 11) of volatile organic compounds (VOCs) collected from the headspace of tomato plant genotypes (susceptible and tolerant) uninfested and infested by *T.* *absoluta *larvae

Compounds	Metabolicclassification	Un-infested		Infested by larvae	
		Susceptibile (S)	Tolerant (T)	Susceptibile (S)	Tolerant (T)
ɑ-pinene	MVA/MEP, monoterpene	41.26 ± 12.95^b^	42.85 ± 16.92^b^	15.05 ± 2.89^c^	**109.65 ± 29.96**^**a**^
Camphene	MVA/MEP, monoterpene	153.03 ± 51.53^a^	**80.86 ± 53.8**^**b**^	18.05 ± 4.36^b^	303.21 ± 87.93^a^
β-pinene	MVA/MEP, monoterpene	7.4 ± 2.8^ab^	3.83 ± 2.94^b^	0.55 ± 0.12^c^	**9.88 ± 3.18**^**a**^
δ3-Carene	MVA/MEP, monoterpene	20.02 ± 7.96^b^	**64.34 ± 9.14**^**a**^	49.29 ± 6.3°	76.02 ± 17.2^a^
ɑ-phellandrene	MVA/MEP, monoterpene	2.55 ± 0.89^a^	**0.28 ± 0.13**^**b**^	0.04 ± 0.01^b^	**1.6 ± 0.6**^**a**^
Myrcene	MVA/MEP, monoterpene	3.16 ± 0.83^b^	2.07 ± 0.42^b^	1.63 ± 0.22^b^	**8.9 ± 2.02**^**a**^
Limonene	MVA/MEP, monoterpene	36.76 ± 11.45^b^	31.42 ± 4.13^b^	21.18 ± 3.36^b^	**81.55 ± 16.53**^**a**^
Eucalyptol	MVA/MEP, monoterpene	13.28 ± 5.13^a^	**1.32 ± 0.78**^**b**^	0.76 ± 0.22^b^	**18.31 ± 5.28**^**a**^
ɣ-terpinene	MVA/MEP, monoterpene	42.83 ± 15.03^c^	**101.92 ± 13.35**^**ab**^	85.93 ± 11.86^b^	**153.01 ± 29.29**^**a**^
Ocimene	MVA/MEP, monoterpene	1.56 ± 0.54^ab^	0.61 ± 0.09^b^	12.05 ± 11.58^b^	**2.73 ± 0.67**^**a**^
Linalool	MVA/MEP, monoterpene	0.05 ± 0.01^ab^	0.08 ± 0.04^b^	0.01 ± 0^c^	**0.29 ± 0.13**^**a**^
(E)-2-hexenal	Fatty acid derivate, aldehyde	5.05 ± 3.72^ab^	**0.34 ± 0.13**^**c**^	0.99 ± 0.59b^c^	**20.71 ± 6.39**^**a**^
cis-3-hexenol	Fatty acid derivate, alcohol	9.89 ± 4.27^a^	**0 ± 0**^**b**^	0.09 ± 0.08^b^	**10.07 ± 3.81**^**a**^
Nonanal	Fatty acid derivate, aldehyde	6.43 ± 3.02^ab^	9.32 ± 5.99b^c^	0.46 ± 0.1^c^	**20.83 ± 9.02**^**a**^
p-cymene	Phenylpropanoid, benzenoid	0.06 ± 0.02^ab^	0.04 ± 0.02^b^	0.04 ± 0.02^b^	**0.09 ± 0.02**^**a**^
Camphor	Phenylpropanoid, benzenoid	5.83 ± 2.7^ab^	0.99 ± 0.75^ab^	0.1 ± 0.05^b^	**4.06 ± 1.49**^**a**^
salicyl-aldehyde	Phenylpropanoid, benzenoid	0.27 ± 0.13^a^	1.79 ± 0.83^a^	0.06 ± 0.04^b^	**0.17 ± 0.07**^**a**^
methyl-salicylate	Phenylpropanoid, benzenoid	0.19 ± 0.06^b^	0.19 ± 0.06^b^	0.22 ± 0.07^b^	**0.59 ± 0.16**^**a**^
	total	349.61 ± 107.88^b^	342.24 ± 86.4^b^	206.5 ± 32.3^b^	**821.65 ± 183.53**^**a**^

^[ab]^Different letters represent significant differences between means at *P* < 0.05, according to Kruskal Wallis test. Significative differences within the two genotypes are highlighted in bold

**Fig. 8:**
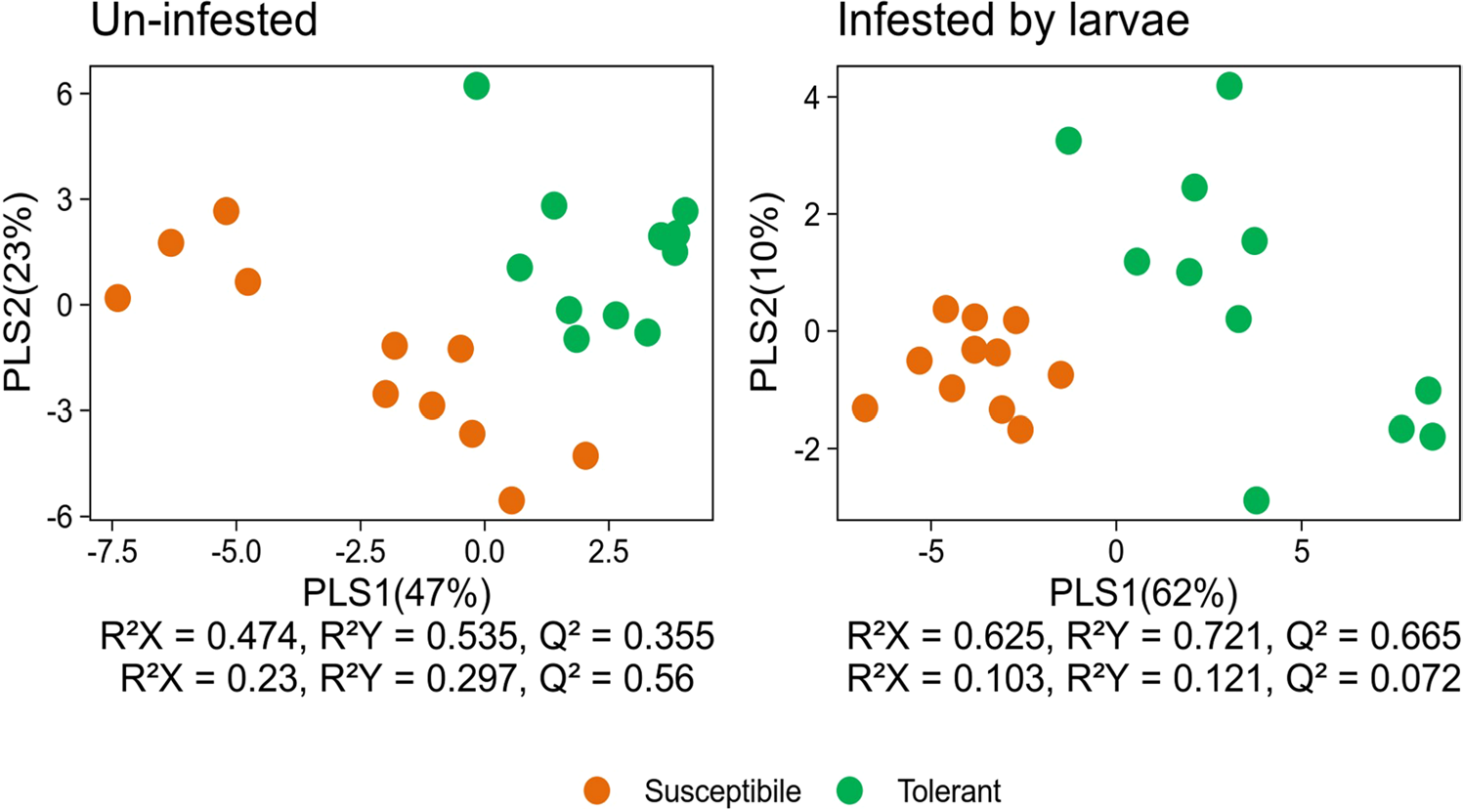
PLS-DA comparison of the volatile compounds emitted by tomato plants genotypes (*S* Susceptible, and *T* Tolerant) uninfested and infested by *T. absoluta* larvae. Score plot of the samples, with the percentage of explained (PLS1-2) variation in parentheses. R^2^X and R^2^Y represent the fraction of variance of the X and Y matrix, respectively, and Q^2^Y represents the predictive accuracy of the model, with cumulative values of R^2^X, R^2^Y and Q^2^Y equating to ~ 1 indicating an effective model

The partial least squares-discriminant analysis (PLS-DA) resulted in two models with six and seven significant components, respectively. The score plots of the VOC emitted by the T and S genotypes showed a high percentage of the explained variation, 70.3% and 72.8%, respectively (Fig. 8). These two models clearly separated the genotypes in both cases (uninfested and infested plants). The influence of the independent variable (VOCs) in explaining the dependent variable (tomato genotypes) was estimated by VIP scores. VOCs with a VIP score greater than 1 are considered pivotal to discriminate plants in the PLS-DA model. Six VOCs (cis-3-hexenol, δ3-Carene, eucalyptol, camphor, ɣ-terpinene and ɑ-phellandrene) and seven VOCs (nonanal, cis-3-hexenol, eucalyptol, camphene, (E)-2-hexenal, β-pinene and camphor) released respectively by uninfested and infested tomato plants, have VIP scores greater than 1.

## Discussion

### Discovery of genomic variation between tolerant and susceptible tomato genotypes to *Tuta absoluta*

The T and S genotypes showed a huge difference in the number of variants, suggesting that they have a divergent genomic background. The distribution of variants along the chromosomes, in both genotypes, showed that SNPs were more frequent in the distal part of chromosomes, which corresponds to regions with higher recombination frequency (Sim et al. 2012) and gene density (The Tomato Genome Consortium 2012). Nevertheless, a dissimilar pattern of variants distribution along the chromosomes, tracing a footprint of different history for the two genotypes, was highlighted. In addition, the identification of contrasting variant pattern between the two genotypes in specific regions of the chromosomes 2, 5, 8 and 12 suggested the presence of important loci involved in response to *T. absoluta*. The identification of private variants led us to identify important differences between the two genotypes. SNPs can have a great impact on the variation of genomes and the phenotypic traits (Shastry 2009) and the presence of private SNPs in the two genotypes could drive a different response to the insect (Mhoswa et al. 2020; Zhou and Jander 2022).

### Genetic variants potentially affecting tomato defenses against *Tuta absoluta*

Several genes with private variants involved in processes associated to plant defense, including stimulus perception, hormone metabolism, cell wall, signaling and secondary metabolism, were identified in the two genotypes. Interestingly, the dissimilar HS regions on chromosome 12 and 2 showed many genes with variants belonging to different classes of receptor kinases that can affect the plant perception of the pathogen. Differences between the two genotypes also regarded the signaling. For example, on the chromosome 2, variants in genes belonging to kinase/phosphatase signaling were found in T genotype. In Solyc02g070260, a phosphatase having a high number of systemin-responsive partners (Ahmad et al. 2019) was affected by several variants. Systemin is a crucial small peptide with important functions in plant wound signaling and defense (Ryan and Pearce 2003). Solyc04g076460, a leucine-rich repeat receptor-like protein involved in plant innate immunity (Zhang et al. 2020b), showed interesting variants in T as well as the kinase SlSERK3B (Solyc01g104970). It is worth mentioning that the silencing of SlSERK3B resulted in enhanced susceptibility to root knot-nematode and non-pathogenic *Pseudomonas syringae* pv. tomato (Peng and Kaloshian2014). In addition, two genes coding for cyclic nucleotide-gated channels (Solyc02g086990 and Solyc02g088560), which have an important role in plant disease resistance and innate immunity (Ma et al. 2009), were affected by variants in T genotype.

Although both genotypes showed variants in the prosystemin gene (Solyc05g051750), the variants were different and had a different effect on the protein and transcript. Solyc05g051750 is constitutively expressed in plant and prompts, also in the absence of wounding, the synthesis of the wound-inducible defensive proteins, whereas its over-expression in antisense orientation blocks the systemic wound signaling (McGurl et al. 1994). Interestingly, transgenic tomato plants overexpressing a truncated prosystemin modulated the expression of defense-related genes against the lepidopteran pest *Spodoptera littoralis* (Molisso et al. 2022).

JA is a lipid-derived phytohormone that plays a crucial role in plants defense against herbivorous insects, and its biosynthesis regulation is very complex (Devoto and Turner 2003). The two genotypes differed for key genes involved in JA synthesis and affected by variants. For example, T-specific variants included the lipoxygenase D TomLoxDon chromosome 3 (Solyc03g122340), which is mainly involved in JA biosynthesis and, when overexpressed, leads to enhanced resistance to insect and necrotrophic pathogen (Yan et al. 2013). On the other hand, the S genotype showed variants in LOXC, which is chloroplast-targeted and generates volatile C6 flavor compounds from both linoleic and linolenic acids (Chen et al. 2021).

T-specific variants were localized in subtilisin-like proteases on chromosome 12, but interestingly a subtilisin-like protease located on chromosome 1 (Solyc01g087850), known to be involved in resistance to the tobacco hornworm *Manduca sexta*, is affected by variants in both genotypes (Meyer et al. 2016). Finally, variants were also found on Solyc02g094040, the ortholog of Arabidopsis *MPL1*, *MYZUS PERSICAE-INDUCED LIPASE 1* (At5g14180), involved in defense against the green peach aphid (Louis et al. 2010).

The cell wall is a complex structure subject to dynamic remodeling that often determines the outcome of the interactions between plants and pathogens (Bellincampi et al. 2014). The presence of variants in cell wall related genes might suggest differences between the two genotypes in cellulose deposition or cell wall remodeling in response to *T. absoluta*. For example, on chromosome 2, the T genotype showed variants affecting a glucan endo-1,3-beta-glucosidase (Solyc02g069700), well known to be implicated in plant defense (Balasubramanian et al. 2012); four CASP-like proteins able to form a mechanical barrier to trap pathogens at the infection site (Lee et al. 2019); three cobra proteins (Solyc02g089115, Solyc02g089120, Solyc02g089130) involved in cell wall composition alteration (Roudier et al. 2005). Variants in the T genotype were also displayed by genes encoding for plant cell wall components involved in defense against pathogens, such as hydroxyproline-rich glycoproteins (Solyc04g076410 and Solyc04g074165) and rhamnogalacturonatelyases (Solyc04g076630, Solyc04g076640, Solyc04g076650, Solyc04g076660) (Deepak et al. 2007).

Several TF genes with a role in biotic stress response, showing variants in the T genotype, were located on chromosome 2. Solyc02g037530, an auxin response factor 8B that promotes JA production (Nagpal et al. 2005; Liu et al. 2014); two zinc finger proteins LSD1, involved in the plant immune system (Solyc02g078270 and Solyc02g069720) (Alves et al. 2014), three WRKY including Solyc02g094270 (Zhang et al. 2020a), Solyc02g088340 *SlWRKY3*, which act as a positive regulator of induced resistance in response to nematode invasion and infection (Chinnapandi et al. 2019), Solyc02g093050 that is ortholog to the Arabidopsis *ATWRKY15*, induced by herbivores (van Aken et al. 2016; Rushton); and the *MYB-SlMIXTA*-like gene (Solyc02g088190) involved in trichome formation (Galdon-Armero et al. 2020; Ying et al. 2020) but also in the regulation and the production and storage of specialized toxic metabolites (Ying et al. 2020). In addition, HD-Zip TF (Solyc08g066500), a negative regulatory of the lignin biosynthesis (Liu et al. 2021), and *ULTRAPETALA* (Solyc12g010755), a regulator of the biotic and abiotic stress response (Tyler et al. 2019), also showed variants in T genotype.

By contrast, S-specific variants affected a *Woolly* gene (Wo), encoding a HD-Zip protein essential for trichome formation (Yang et al. 2011) and Solyc12g005830, *SlHDZIV8*/similar to the *HDG2*, *HOMEODOMAIN GLABROUS 2* gene, participating in trichome development in Arabidopsis (Marks et al. 2009). S-specific variants were also identified in genes involved in the trichome formation: Solyc10g077070, a gene characterized by Fonseca et al. (2022) as *HAIRPLUS* (*HAP*), which controls glandular trichome density in tomato plants, and the *CUTIN DEFICIENT2* (Solyc01g091630), well known gene that regulates cuticle deposition and the formation of glandular type-VI trichomes. *CUTIN DEFICIENT2* variant alleles promoted reduced trichome density and lower volatile terpene production (Nadakuduti et al. 2012).

### Identification of genes with variants involved in volatile organic compounds production

Many genes with variants were identified in secondary metabolism and were involved in the biosynthesis of VOCs, including phenylpropanoids, flavonoids and terpenoids, which play a key role in direct or indirect defense (War et al. 2012; Cheynier et al. 2013; Kessler 2017; Ameye et al. 2018; Erb and Kliebenstein 2020). In the cultivated tomato, acyl sugars, flavonoids, and terpenes are major secondary compounds produced in type I, IV and VI gland bearing trichomes in response to herbivore attack (Schilmiller et al. 2009; McDowell et al. 2011; Kang et al. 2014; Bergau et al. 2015; Balcke et al. 2017).

Caffeoyl-CoA O-methyltransferase (CCoAOMT) is essential in lignin biosynthesis, an important barrier that protects against pests and pathogens (Liu et al. 2018). Notably, a CCoAOMT (Solyc02g093230) affected by variants in T genotypes was located in a QTL region implicated in resistance against pathogens and herbivores (Vosman et al. 2018, 2019).

In both genotypes, the identification of genes involved in acylsugar biosynthesis and export, affected by variants, could suggest a diversity in the acylsucrose types and in acylglucose abundance. Genes involved in acylsugars biosynthetic pathway are well known to be activated in the trichomes of solanaceous plants to combat herbivores and pathogens (Fobes et al. 1985; Kroumova et al. 2016; Moghe et al. 2017). Interestingly, T variants were found in Solyc03g121540, encoding for ACYLSUCROSE FRUCTOFURANOSIDASE 1 (ASFF1), a trichome gland cell–expressed invertase that cleaves the glycosidic bond of P-type acylsucroses to generate acylglucoses (Leong et al. 2019). It is worth to note that *ASFF1* gene is located in the QTL AG3.2 region at the bottom of chromosome 3 (Leckie et al. 2013; Leong et al. 2019). In addition, the presence of variants in genes involved in the biosynthesis of acylsugar isoC5-CoA acyl chain precursor could lead to a different use of isoC5-CoA as a donor, leading to an accumulation of diverse sets of acylsucrose structures (Fan et al. 2019). The S genotype could be affected in acylsugar export due to variants in the ABC transporter Solyc03g005860, previously associated with acylsugar exudation (Mandal et al. 2020). Acylsugar transport could be critical in determining how much acylsugar is produced and secreted, with significant consequences on plant defense. Instead, the T variants discovered in Solyc02g093180 could suggest an impact on the trichome production of acylsugars. This gene, encoding for an acyltransferase involved in acyl sugar biosynthesis (Fan et al. 2016), is located in a QTL region at the bottom of the chromosome 2 (Vosman et al. 2019), related to the presence of trichome type IV (Wf-1).

Among the plant secondary metabolites involved in biotic stress tolerance, terpenoids are the most diverse (Gershenzon and Dudareva 2007; Coppola et al. 2018; Boncan et al. 2020). The presence of variants in genes involved in the biosynthesis of different classes of terpenes in both genotypes could drive a metabolic diversity that can affect the response to *T.* *absoluta*. For example, T unique variants in the terpene synthase Solyc02g079840, located on the chromosome 2, were found. Such gene is involved in the (−)-α-terpineol biosynthesis, a compound with insecticidal properties (Khaleel et al. 2018) as well in 7-deoxyloganetin glucosyltransferases, enzymes involved in the synthesis of the monoterpene-derived compounds, called iridoids glycosides, that have a potential role in defense against herbivores (Puttick and Bowers 1988; Biere et al. 2004; War et al. 2018). In addition, both genotypes had variants in genes involved in the production of lupeol and amyrin, which are pentacyclic terpene with a major role as precursors for specialized triterpenoid metabolites, involved in plant defense and development (Cárdenas et al. 2019). T showed also specific variants in a gene producing caryophyllene that functions as a signal in the plant defense against herbivores (Köllner et al. 2008). Finally, a BHLH transcription factor (Solyc09g083360) involved in terpene biosynthesis and resistance against cotton bollworm and *B. cinerea* (Cao et al. 2022) and Solyc12g09990, scarecrow-like 3 (*SlSCL3*) involved in the production of monoterpenes and sesquiterpenes (Yang et al. 2021) showed variants in S genotype.

### Volatile phenotyping

VOCs play an important role in the interactions of tomato genotypes with insect pests and also with their antagonists (Gontijo et al. 2019) and some of the compounds identified could be considered markers to separate genotypes resistant to *T. absoluta* infestation.

All compounds identified in the present work are in accordance with literature data concerning the VOC released by tomato plants upon herbivore feeding (i.e. *T. absoluta* larvae) (Proffit et al. 2011; Anastasaki et al. 2015, 2018; Catola et al. 2018; Milonas et al. 2019; Subramani et al. 2021; Ayelo et al. 2022; Deletre et al. 2022; Miano et al. 2022). In particular, we recorded a blend of volatiles emitted by tomato plants infested by *Tuta* larvae quantitatively different with respect to undamaged control plants as already reported (Anastasaki et al. 2015, 2018; Milonas et al. 2019; Ayelo et al. 2022). We highlighted an increased level of δ3-carene, ɣ-terpinene, ocimene and methyl-salicylate in both S and T genotypes plants and a higher level of ɑ-pinene, β-pinene, linalool, cis-3-hexenol and nonanal in T genotype plants infested by *T. absoluta* larvae compared to uninfested ones (Anastasaki et al. 2015, 2018; Milonas et al. 2019; Ayelo et al. 2022). Almost all compounds identified in this study are recognized by *T. absoluta* antennae (Anastasaki et al. 2018; Miano et al. 2022) and some of them have been recently evaluated for their possible use as repellents towards adult pests (Essoung et al. 2020; Miano et al. 2022). For example, α-pinene, β-pinene are oviposition deterrent for several pests of stored food products (Regnault-Roger and Hamraoui 1995; Ferrarini et al. 2008; Chaubey 2012) and the potato tuber moth, *Phthorimaea operculella* (Sharaby et al. 2009). In line with these evidences, we found a significant reduction of α-pinene (− 63.5%), β-pinene (− 92.6%) released by S genotype plants following larval feeding. Conversely, we recorded a significant increase of these two compounds (+ 155.9% and 158.0%, respectively) in the blend released by T genotype plants upon larval feeding. Similarly, in T we found a significantly higher release of compounds with repellence proprieties toward *T. absoluta* adults (Essoung et al. 2020; Miano et al. 2022) such as camphene, eucalyptol and camphor these (+ 275.0%, + 1287.1% and + 310.1%, respectively). Changes in the level of these marker VOCs could be associated with the tolerance/susceptibility to the tomato leaf miner.

## Conclusion

Here, the identification of SNP/InDels from expressed genes, in contrasting tomato genotypes for the response to *Tuta absoluta* infestation, allowed us to identify chromosome regions with a highly dissimilar pattern. Interesting, variants in genes involved in the defense response to herbivory and in transcription factors controlling a series of genes responsive to stress were highlighted. They result good candidates to be explored for improving our understanding of tolerance to insect pests in tomato. Finally, genes related to different biochemical classes of VOCs, which play a key role in direct or indirect defense, were affected by several variants as well as the production of phenylpropanoids and benzenoids, terpenoids compounds both from uninfested and infested plants underlined significant differences. All these findings are a valuable resource for tomato breeding aiming to develop plants tolerant to *T. absoluta*. Future work will confirm whether these variants detected in the analyzed genes are responsible for the different response to *T. absoluta*.

### *Author contribution statement*

DD was centrally involved in gene functional annotation, data interpretation and in manuscript writing. AG and CGA were involved in variant identification and annotation. PC and EG performed infestation experiments, VOC collection, analysis and interpretation. GC and MM performed VOC analysis and interpretation. MRE conceived the study and was mainly involved in data interpretation and in manuscript writing. All authors read and approved the final manuscript.

## Supplementary Information

Below is the link to the electronic supplementary material.

Supplementary file1 (PDF 215 KB)

Supplementary file2 (XLSX 4465 KB)

## Data Availability

The Illumina sequence data are accessible at NCBI's Gene Expression Omnibus (http://www.ncbi.nlm.nih.gov/geo) with accession number GSE159085. Variant Data are reported in additional supporting files.

## Abbreviations

InDel: Insertions/deletion
HS: SNPs hotspot
JA: Jasmonic acid
SNP: Single nucleotide polymorphism
S: Susceptible tomato variety
T: Tolerant tomato variety
TF: Transcription factor
VOC: Volatile organic compound
