# Supplementary material for: Genomic and metabolic profiling of two tomato contrasting cultivars for tolerance to Tuta absoluta
Source: Planta. 2023 Jan 28;257(3):47. doi: 10.1007/s00425-023-04073-8 (PMC9884263; doi:10.1007/s00425-023-04073-8)
Supplement: Supplementary file 1 — Supplementary file1 (PDF 215 KB) [file 425_2023_4073_MOESM1_ESM.pdf]

Supplementary Figures

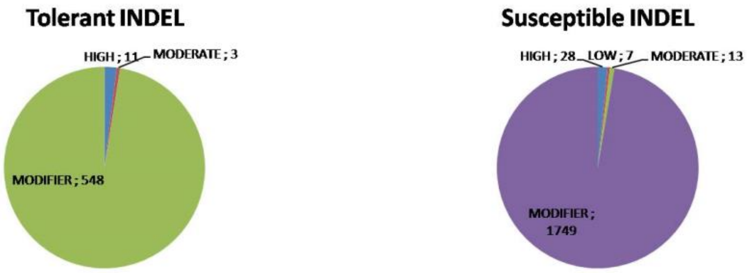

Supplementary Fig. S1 Number of InDels classified as high, moderate, modifier and low effect in tolerant and susceptible

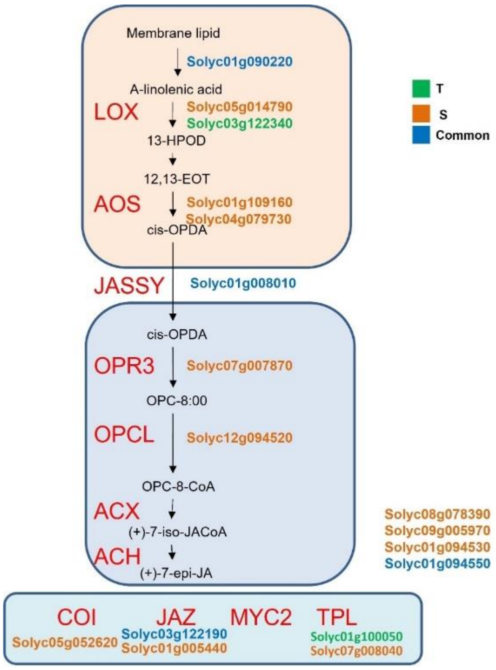

Supplementary Fig. S2 Genes affected by variants in jasmonic acid biosynthesis and signaling. In green are indicated Tolerant-specific genes, in orange are indicated Susceptible-specific genes and in blue are indicated common genes

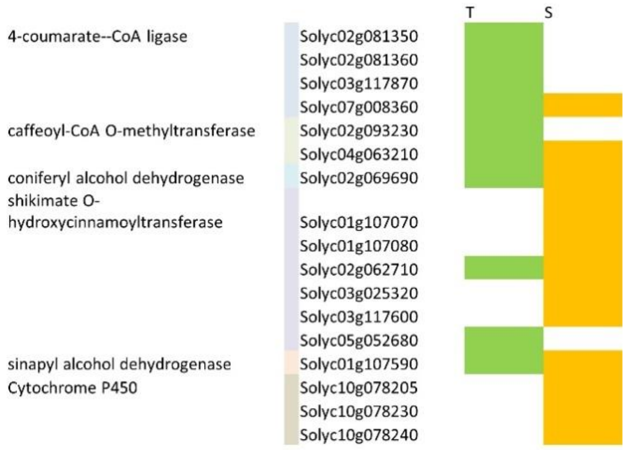

Supplementary Fig. S3 Genes involved in phenylpropanoid biosynthesis affected by variants. T = tolerant, S = susceptible

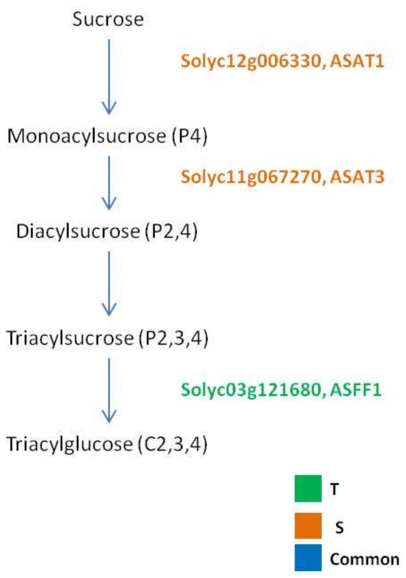

Supplementary Fig. S4 Genes affected by variants in the Phase 2 of acylsugar production
